# Temporal Distinctiveness in Task Switching: Assessing the Mixture-Distribution Assumption

**DOI:** 10.3389/fpsyg.2016.00251

**Published:** 2016-02-24

**Authors:** James A. Grange

**Affiliations:** School of Psychology, Keele UniversityKeele, UK

**Keywords:** task switching, decay, interference, computational model

## Abstract

In task switching, increasing the response–cue interval has been shown to reduce the switch cost. This has been attributed to a time-based decay process influencing the activation of memory representations of tasks (task-sets). Recently, an alternative account based on interference rather than decay has been successfully applied to this data (Horoufchin et al., 2011a). In this account, variation of the RCI is thought to influence the *temporal distinctiveness* (TD) of episodic traces in memory, thus affecting their retrieval probability. This can affect performance as retrieval probability influences response time: If retrieval succeeds, responding is fast due to positive priming; if retrieval fails, responding is slow, due to having to perform the task via a slow algorithmic process. This account—and a recent formal model (Grange and Cross, 2015)—makes the strong prediction that all RTs are a mixture of one of two processes: a fast process when retrieval succeeds, and a slow process when retrieval fails. The present paper assesses the evidence for this *mixture-distribution* assumption in TD data. In a first section, statistical evidence for mixture-distributions is found using the fixed-point property test. In a second section, a mathematical process model with mixture-distributions at its core is fitted to the response time distribution data. Both approaches provide good evidence in support of the mixture-distribution assumption, and thus support temporal distinctiveness accounts of the data.

## Introduction

The task switching paradigm is a popular method for studying cognitive control (Kiesel et al., 2010; Vandierendonck et al., 2010; Grange and Houghton, 2014). Within this paradigm, participants are required to perform simple cognitive operations on multi-valent stimuli; for example, participants might be presented with numerical stimuli, and be asked to judge either whether the stimulus is odd/even (task A), or whether the stimulus is lower/higher than 5 (task B). Participants know which task to perform as they are presented with a cue informing them which task is relevant for the current trial (e.g., the word “magnitude” might cue the lower/higher task). It is a consistent finding that switching tasks (e.g., A–B) induces a performance cost in the form of increased response time (RT) and error rates compared to repeating the same task (e.g., A–A); this *switch cost* has become the focus of intense research in an attempt to understand its underlying causes.

More recently, research has started to focus on the nature of the memory representations used to perform these tasks. As the stimulus is usually not informative as to which task needs to be performed, a so-called “task-set” needs to be formed in working memory, which can be considered a collection of programmable task parameters (such as attentional bias etc.) critical to task performance (Logan and Gordon, 2001); this task-set must be changed when the task changes. A central goal for efficient cognitive control is to ensure that the relevant task-set is the most active among all competitors (e.g., Altmann and Gray, 2008). Therefore, an understanding of the dynamics of activation of task-sets is central to a complete understanding of the challenge facing cognitive control.

Evidence from the task switching paradigm has provided several lines of evidence suggesting that activation levels of task-sets decay passively as a function of time. For example, Meiran et al. (2000) varied the temporal separation between successive tasks via the response–cue interval (RCI), the time between the response to one task and the onset of the cue for the next task. These authors reported reduced switch costs at longer RCIs; this finding is in perfect accord with a decay account for two reasons: first, on task repetition trials, the previous task's activation will have had more time to decay at longer RCIs, meaning its ability to prime performance on the current trial will have reduced (leading to slower RTs); secondly, on task switch trials, the activation levels of the previous (now irrelevant) task-set will have decayed, inducing less proactive interference on the current trial (thus resulting in faster RTs). The net result is a reduced switch cost. It should be noted that several studies have found task repetition RTs slow as a function of RCI, the evidence for the predicted speeding of switch RT is less clear. In fact, several studies have found no evidence for RCI affecting switch RTs in the predicted direction (Meiran et al., 2000; Altmann, 2005; Horoufchin et al., 2011a). However, slowing of repetition RTs with increasing RCI remains an important finding supporting decay accounts.

However, this decay account was recently challenged by Horoufchin et al. (2011a) who manipulated RCI on a trial-to-trial basis between a short- (e.g., 50 ms) and a long-RCI (1000 ms); they found that the reduction of switch cost was not a function of *absolute* time of the RCI (e.g., between the previous trial n–1 and the current cue at n), but rather was a function of the ratio of the current RCI to that from the previous trial (between trials n–2 and n–1). Specifically, the switch cost was only reduced at longer RCIs when the previous RCI was short; when the previous RCI was also long, switch costs were not reduced. These effects were primarily localized to task repetition RT; task switch RT was not influenced by RCI or the RCI-ratio.

**Figure 1:**
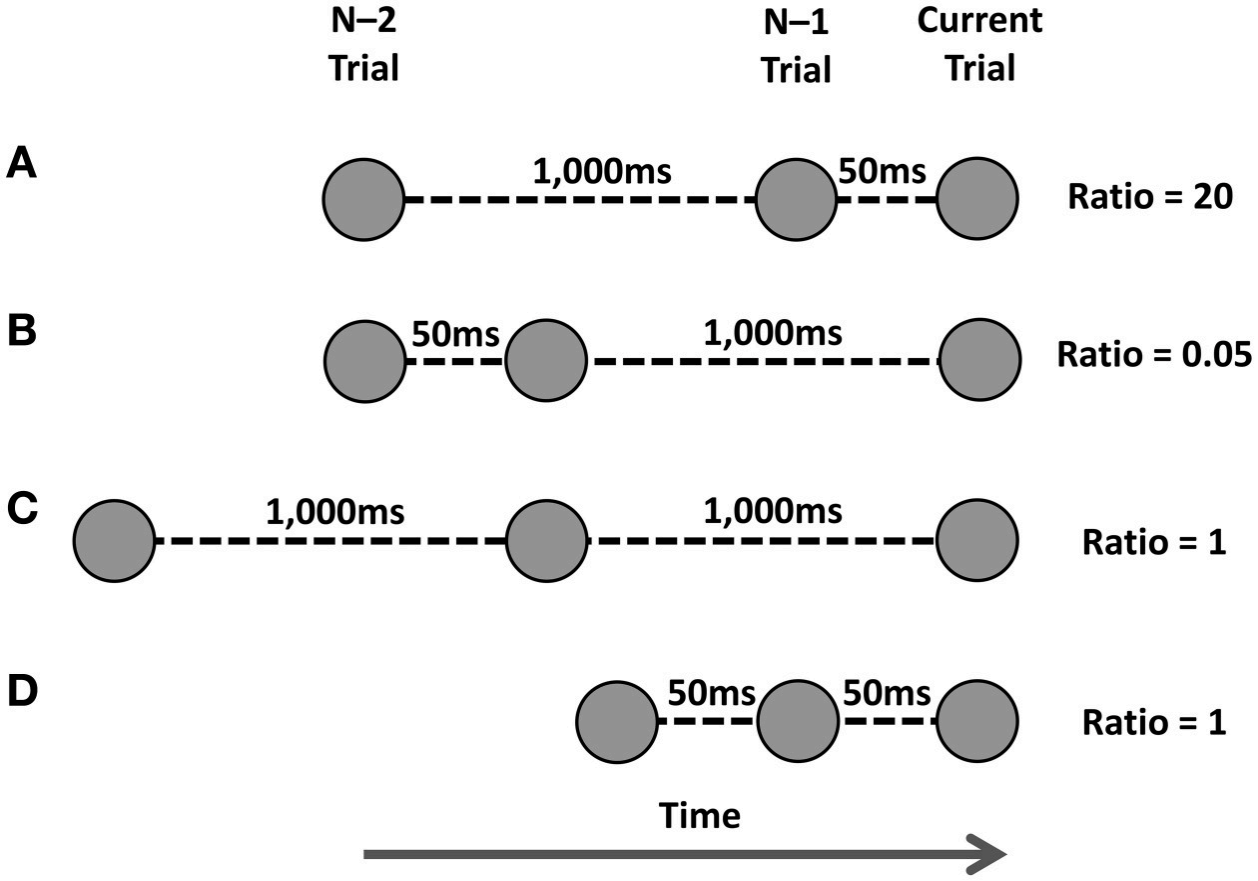
**Various manipulations of temporal distinctiveness as used by Horoufchin et al. (2011a,b)**. Task instances are represented by circles, and time runs from left to right. The current trial's cue is represented by the far-right circle; the episodic trace of the previous task is the middle circle, and the episodic trace of the task from two trials ago is the far-left circle. The RCI between each trace is shown for each row. The ratio is calculated as RCI(n-2:n–1) / RCI(n–1:current trial). See text for more details. **(A)** Ratio of 20. **(B)** Ratio of 0.05. **(C,D)** Ratio of 1.

Horoufchin et al. (2011a) proposed these findings supported an *interference* account of RCI effects, and were not compatible with a decay account. Relating to a temporal ratio model of serial memory (SIMPLE Brown et al., 2007), the authors suggested that when presented with a cue on the current trial, the cognitive system engages in a retrieval attempt of the previous episodic memory trace of the task-set associated with this cue; such retrieval attempts have been shown by Brown et al. (2007) to be influenced by the *temporal distinctiveness* (TD) of the episodic trace. TD refers to the degree to which the targeted episodic trace is distinguishable in memory from competing memory traces presented within a similar time-window to that of the target trace. Traces that are clustered closely in episodic memory will not be distinguishable, and thus have low TD and will have low probability to be retrieved. The sequential varying of the RCI was suggested to affect the TD of the targeted episodic trace of the task-set in memory. TD is operationalized in relation to the RCI-ratio, which is the ratio of the RCI(n–2:n–1) / RCI(n–1:current trial). See Figure 1 for how various RCI-ratios are calculated.

When RCI-ratio is high, so too is TD, which means there is a high probability the episodic trace can be retrieved; successful retrieval of the task-set primes performance, leading to a fast RT. When the RCI-ratio is low, so too is TD, which lowers the probability of successful retrieval of the task-set. When retrieval fails, the task must be performed via a slower, algorithmic route, leading to a slower RT (see Logan, 2002, for a similar proposal). Intermediate RCI-ratios lead to intermediate levels of TD; therefore, a proportion of these trials will have successful retrieval, and some trials will have failed retrieval.

**Figure 2:**
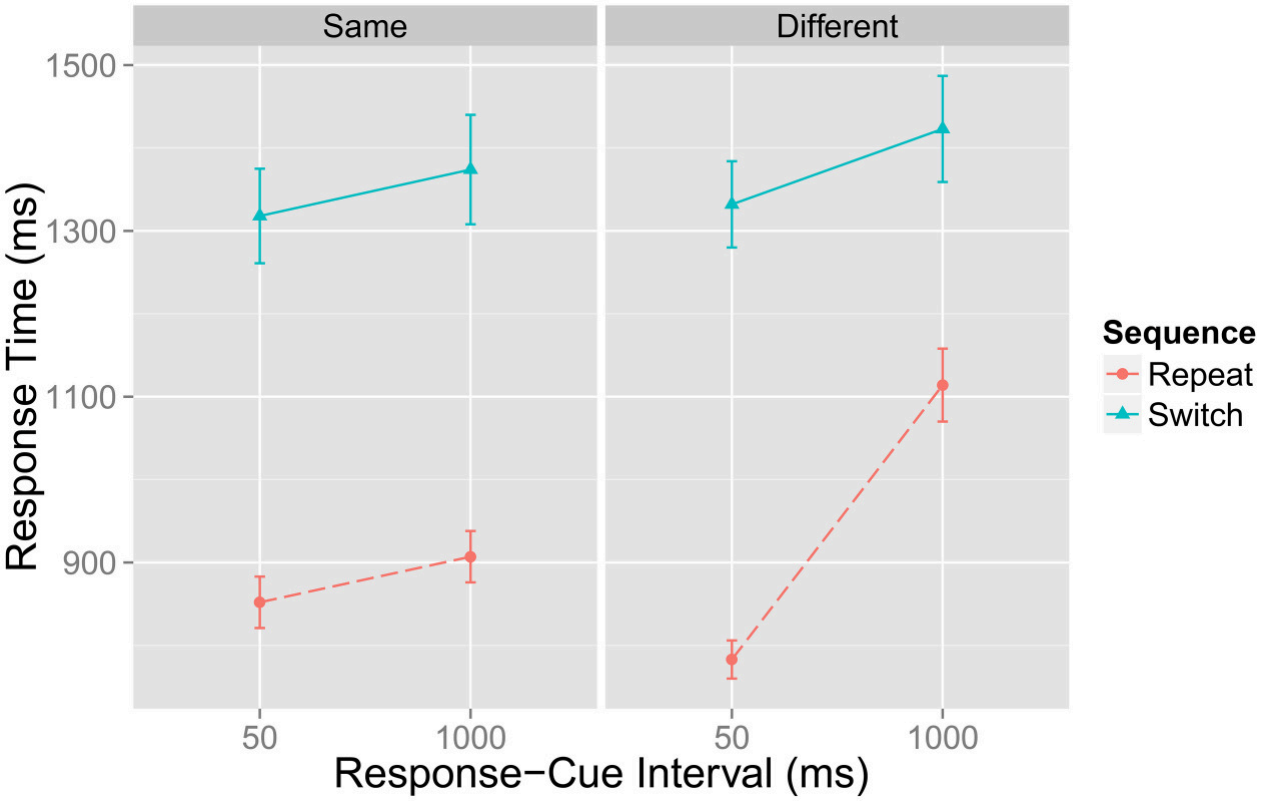
**Data from Grange (in revision) showing mean response time in milliseconds (ms) for task repetition and switch trials as a function of the current response–cue interval (x-axis) and whether the RCI is the same or different from the previous trial**. Error bars denote ±1 standard error around the mean.

Figure 2 represents a portion of data reported by Grange (in revision)—a brief overview of the pertinent method details and analysis are presented in Appendix A—that replicates the main findings from Horoufchin et al. (2011a); focussing on repetition RTs, increasing RCI from 50 to 1000 ms has negligible effect on RTs when the RCI was the same as the previous trial (e.g., these data points relate to D and C in Figure 1); this is because both of these conditions have an identical RCI-ratio of 1, and hence have the same TD. RCI has a considerable effect on repetition RT when the RCI has changed from the previous trial: RT is very fast when the RCI is 50 ms, as this condition has a very high RCI-ratio, hence high TD (Figure 1A); RT is very slow when the RCI is 1000 ms, as this condition has a very low RCI-ratio, hence low TD (Figure 1B).

Note that a decay account cannot explain this data, as it would predict identical slowing of repetition RTs at longer RCIs *regardless* of the RCI on the previous trial (whether it was the same or different to that of the previous trial). Thus, this data presents a strong challenge to the decay account of task-sets.

## The current study

Given the important of the dynamics of task-sets for the development of theoretical accounts of task switching (e.g., Schneider and Logan, 2005; Altmann and Gray, 2008), the temporal distinctiveness account provides an important challenge to our understanding of cognitive control in task switching. Given this importance, Grange and Cross (2015) developed a basic mathematical model of TD effects using SIMPLE (Brown et al., 2007) to calculate the TD for a range of RCI-ratios, and in turn to predict mean RT for a range of RCI-ratios.

In this model—using the mathematics of SIMPLE—the RCI-ratio can be translated in to a measure of the *Distinctiveness* of an episodic trace. Distinctiveness is proportional to the probability of retrieving this episodic trace, denoted *p*(retrieval). As per the verbal theory of Horoufchin et al. (2011a) it is assumed that if retrieval succeeds, the response for that trial will be sampled from a fast RT process, with mean μ_*Fast*_; if retrieval fails, the response for that trial will be sampled from a slow RT process, with mean μ_*Slow*_. Therefore, trials with high RCI-ratio (e.g., 20) will have high TD, and therefore high *p*(retrieved), meaning many trials will be sampled from the fast RT process; trials with low RCI-ratio (e.g., 0.05) will have low TD, and hence low *p*(retrieved), meaning many trials will be sampled from the slow process. See Appendix B for an overview of this model.

In the model, mean RT for a given RCI-ratio is predicted by

(1) $$\begin{array}{c} RT=p(retrieved)\times \mu_{Fast}+[1-p(retrieved)]\times \mu_{Slow}. \end{array}$$

Importantly, this model therefore assumes that all RTs are being sampled from one of only two processes: a fast and a slow process. Therefore, trials with intermediate RCI-ratios (e.g., 1) will have intermediate TDs, and therefore intermediate *p*(retrieved); this means that intermediate RCI-ratio RTs will be a mixture of RTs from the fast process and RTs from the slow process, with the exact proportion of mixture related to *p*(retrieved).

The purpose of the present study is to assess in more detail the validity of this key prediction of the temporal distinctiveness account: that intermediate RCI-ratio RTs are a mixture of samples from a fast process and a slow process. I call this assumption the *mixture-distribution* assumption.

I approach this assessment in two ways. In the first, I use a statistical test—the fixed-point property test—to assess the presence/absence of evidence for intermediate RCI-ratios being a mixture of a fast and a slow distribution. Then, in a next step I extend the model of Grange and Cross (2015) to predict whole RT-distributions, which allows a direct test of the mixture-distribution assumption.

### Switch trials

Note that the current paper does not attempt to model switch RT performance. This is for several reasons. One is that there is evidence that RCI does not affect switch RT (Meiran et al., 2000; Altmann, 2005; Horoufchin et al., 2011a). Second, the temporal distinctiveness account of Horoufchin et al. (2011a) mostly considers task repetition performance because the RCI-ratio did not influence switch trials. As such, the formal model of Grange and Cross (2015) only modeled repetition RTs. As the purpose of the present paper is to test further the main assumptions of the account of Horoufchin et al. (2011a) and the formal model of Grange and Cross (2015), the current work continues to focus on repetition RT. A complete model would of course have to account for switch trial performance, too, but as these trials appear unaffected by temporal distinctiveness they are less interesting for the aims of the current paper. I leave discussion of this issue to the General Discussion.

## Assessing the presence of mixture-distributions

Van Maanen et al. (2014) introduced a method—the “fixed-point” property test—for statistically testing the presence of a mixture-distribution. If distribution *z* is a mixture of two base distributions *x* and *y*, then the fixed-point property states that the probability density for distribution *z*, *f*_*z*_, is a weighted sum of the probability densities of the other two distributions *f*_*x*_ and *f*_*y*_. Put simply, this implies that there exists a particular response time *t* for which the probability of providing such a response is identical for all three distributions; that is *f*_*x*_(*t*) = *f*_*z*_(*t*) = *f*_*y*_(*t*).

**Figure 3:**
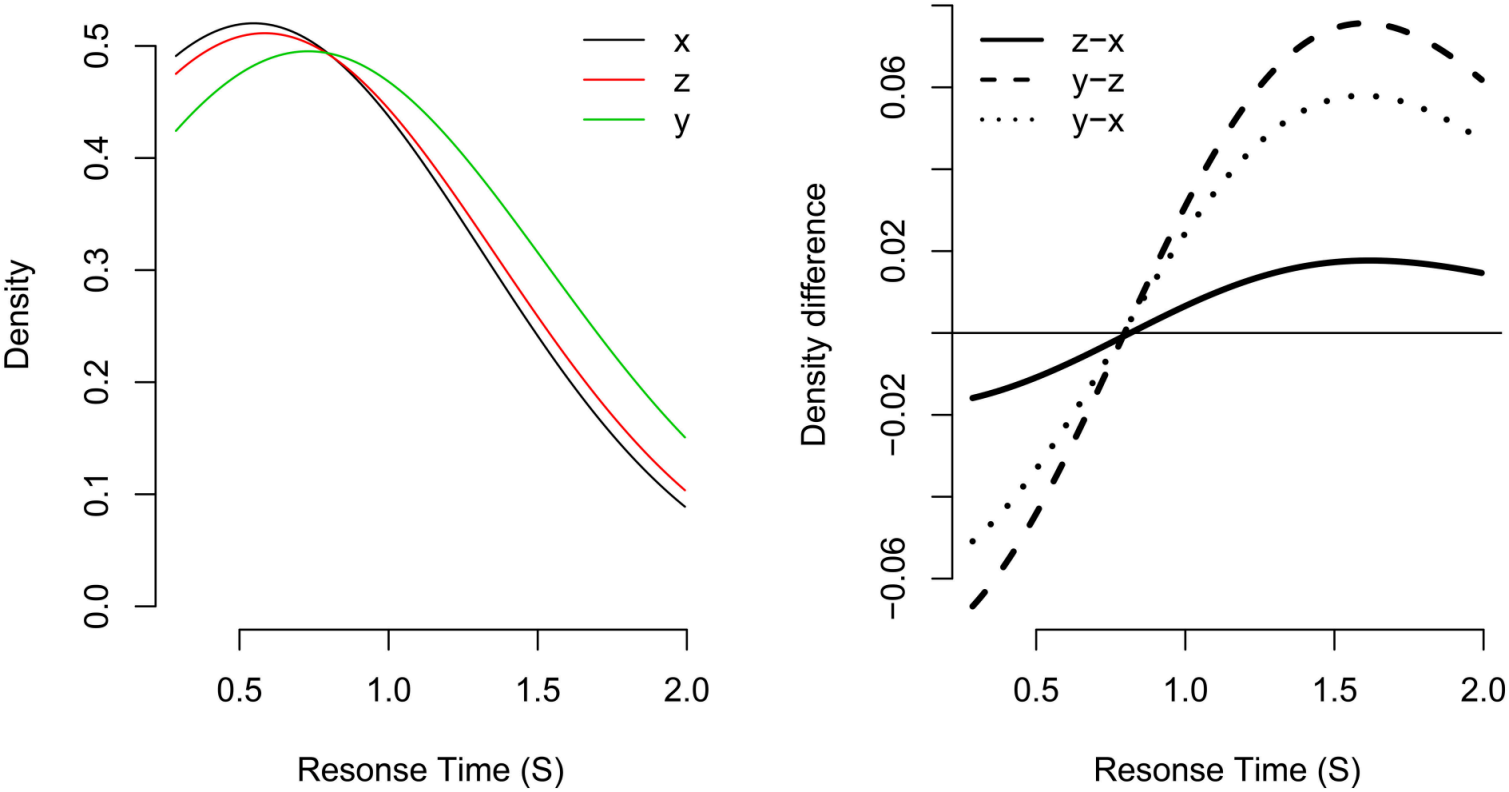
**Example of the fixed-point property in three distributions**. Distribution *z* is a mixture of distributions *x* and *y* with mixture probability *p* set to *p* = 0.7. The left panel shows the probability densities for each distribution. The right panel shows the density difference between different density functions. The distributions share a common crossing point at about 0.850 s.

For example, consider the left panel of Figure 3. Here, three response time distributions have been simulated: *x* is a fast RT distribution, and *y* is a slow RT distribution. Distribution *z* was simulated as being a mixture of distributions *x* and *y* with mixture probability *p* set to *p* = 0.7.

As can be seen, the density functions of all three distributions share a common “crossing point,” the RT at which all density functions are equal (i.e., where *f*_*x*_(*t*) = *f*_*z*_(*t*) = *f*_*y*_(*t*)). The crossing point is made clearer by taking successive subtractions of pairs of density functions (see the right panel of Figure 3). Zero in this plot represents a density difference of zero (i.e., where the two density functions are equal). For example, the solid line represents the density difference of *f*_*z*_ - *f*_*x*_. If the distributions share a common crossing point, the density differences for all pairs of distribution differences will cross zero at a similar response time. This is the case in the current plot, where the three distributions share a common crossing point of about 0.850 s.

### Application to the data

The task repetition data from Figure 2 was assessed for the presence of a fixed-point property. In this test, the RCI-ratio of 20 can be considered the “fast” distribution, and the RCI-ratio of 0.05 can be considered the “slow” distribution. The critical question is whether intermediate RCI-ratios of 1 are a mixture of the fast and the slow distribution. For this test, I collapsed the 50–50 and 1000–1000 data, as both represent an RCI-ratio of 1. The mean difference between these two conditions was 54.8 ms, but was not statistically significant, *t*_(24)_ = 1.7496, *p* = 0.09. The Bayes factor for this test was *BF*_01_ = 1.26, which slightly favors the null of no difference (although this is not decisive).

**Figure 4:**
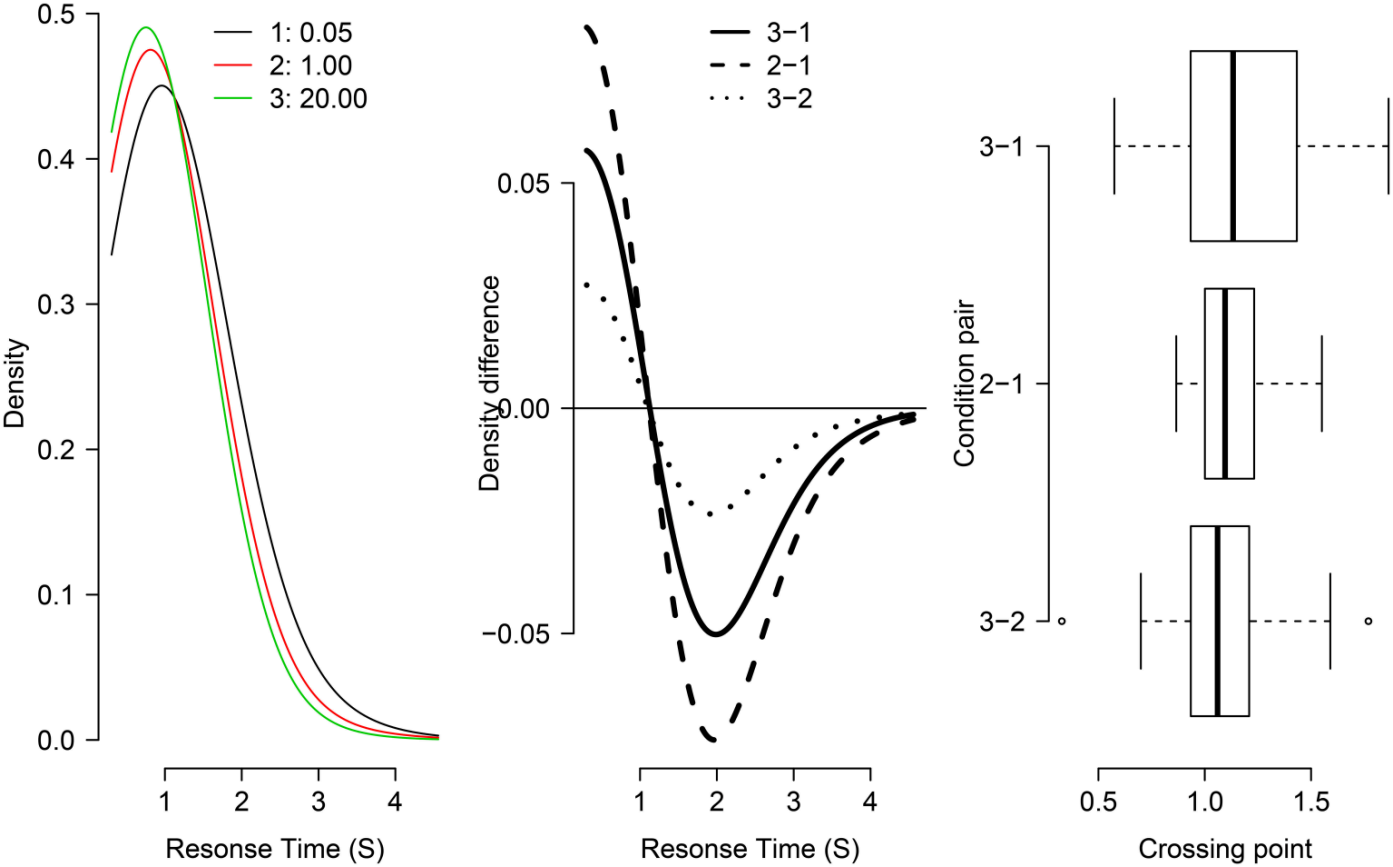
**Test of the fixed-point property in three distributions from Grange (in revision)**. The left panel shows the individual density functions for each condition of RCI-ratio (1 = 0.05, 2 = 1, 3 = 20). The middle panel shows “crossing points”: the subtraction of pairs of density functions (indicated in legend). The right panel shows box-plots for the distribution of crossing points for individual participants in the study.

Data were trimmed before testing. Correct RTs were trimmed to retain RTs slower than 150 ms, and RTs faster than 2.5 SD above each subjects' mean RT. The data was then assessed for the presence of a fixed-point by passing it to the fp function provided in the form of R code from Van Maanen et al. (2014). This takes the individual participants' RT data as input. Visual representation of the fixed-point assessment is shown in Figure 4.

As shown in the left and center panels of Figure 4, the three group-averaged RT distribution functions share a common crossing point at just over 1 second. The right panel of Figure 4 shows box-plots of the distribution across individual participant data of crossing points for each pair. There is considerable overlap in the box-plots suggesting no difference between condition pairs of crossing point (supporting the fixed-point property). Together, this visual inspection provides good evidence that the RT function for the RCI-ratio of 1 is a weighted mixture of the “slow” RT distribution (where RCI-ratio = 0.05) and the “fast” RT distribution (where RCI-ratio = 20), as predicted by the temporal distinctiveness model (Grange and Cross, 2015).

To assess statistical support for the common crossing point, a one-way ANOVA was conducted on the three levels of crossing-point pairs shown in the right panel of Figure 4. The dependent variable in this analysis—shown in the x-axis of that Figure—is the crossing point in seconds for the density difference. This analysis was not statistically significant [*F*_(2, 48)_ = 1.25, **p** = 0.295], which provides no reason to reject the null hypothesis of a common crossing point. However, to confirm the statistical *presence* of a common crossing point requires the acceptance of a null hypothesis which cannot be achieved via standard null hypothesis significance testing. Therefore, a default Bayesian ANOVA (Rouder et al., 2012) was conducted on the same data, which produces a Bayes Factor; the Bayes Factor—denoted *BF*_01_—assesses the evidence in favor of a model assuming a common crossing point (i.e., a “null” model) compared to a model assuming multiple crossing points (i.e., an “alternative” model). The Bayes Factor for this data was *BF*_01_ = 3.354, suggesting the data are 3.4 times more likely under the model assuming a common crossing point. Together, these statistical analyses converge on the conclusion that the RT distributions share a common crossing point, and as such can be considered support for the mixture-distribution assumption.

## A mixture-distribution model of temporal distinctiveness effects

The previous section provided statistical support for the presence of a mixture-distribution for intermediate RCI ratios (i.e., intermediate levels of temporal distinctiveness). This is in agreement both with the verbal theory regarding TD effects (Horoufchin et al., 2011a) and its formal implementation (Grange and Cross, 2015), which explains performance across RCI-ratios. In this section I develop a mathematical process model to predict RT distributions of RCI-ratio data. The fitting of whole RT distributions presents an important advance over the model of Grange and Cross (2015), as it allows a direct test—rather than a relatively indirect test from fitting mean RTs—of that model's core assumption: that intermediate TD performance is a weighted mixture of “retrieved” and “not-retrieved” processing modes, which lead to fast and slow RTs, respectively.

In this section, I provide a schematic overview of the assumptions of the model. The mathematical details of the model are in Appendix C.

### Overview of the model

**Figure 5:**
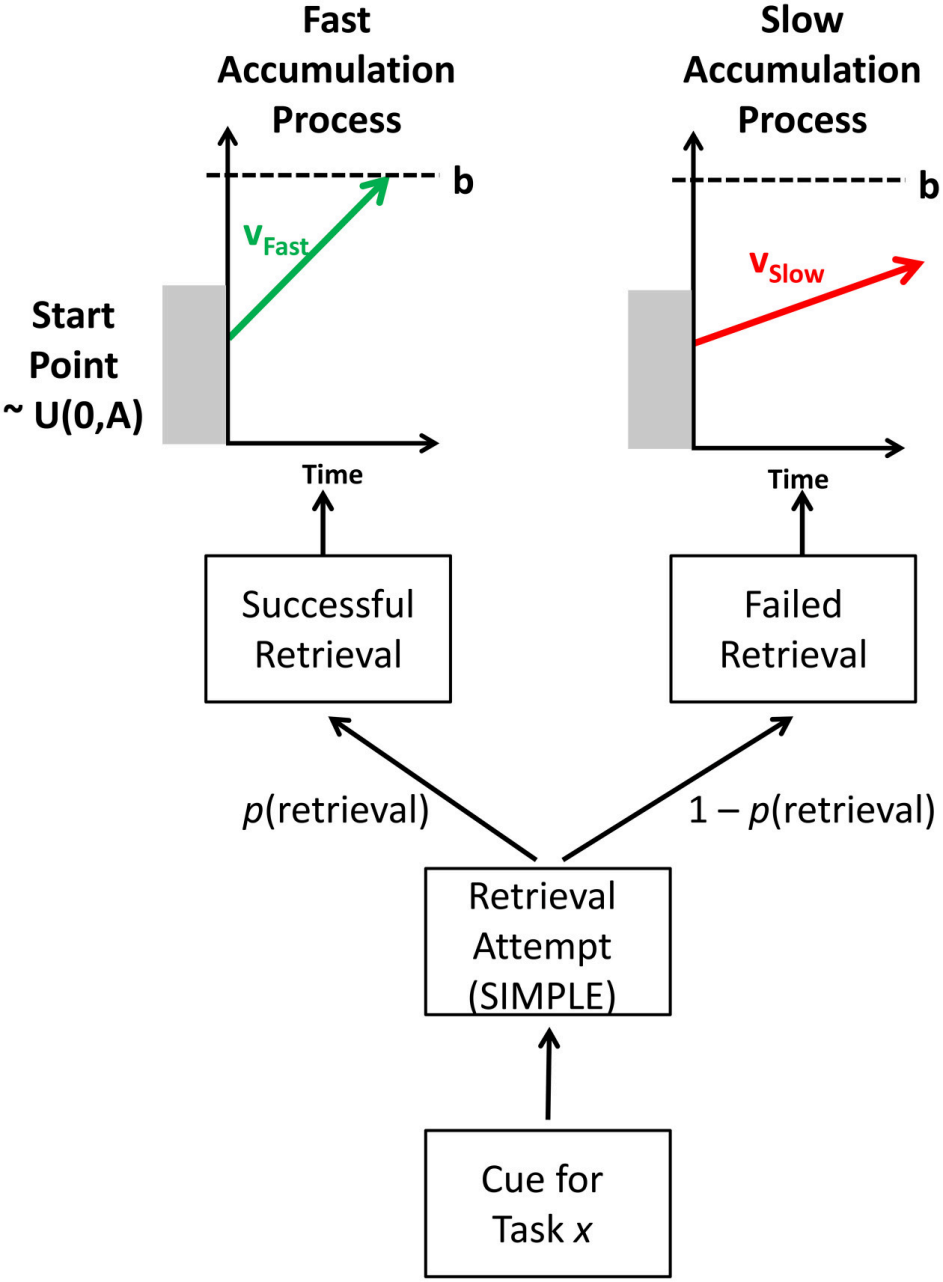
**Overview of the retrieval processes in the model**.

The model describes two stages to performance: an episodic retrieval attempt of the target trace, and an evidence accumulation process that generates a response; the rate of evidence accumulation toward a response depends on whether the trace was retrieved or not, with accumulation rates being faster for successfully retrieved traces. The main processing stages in the model are shown in Figure 5.

#### Episodic retrieval

The model assumes that, when presented with a task cue on a task repetition trial, the cognitive system attempts to retrieve the most recent episodic trace of this task (which was from trial n–1). The success of this retrieval attempt is influenced by the temporal distinctiveness of the episodic trace (at n–1) from a distracting episodic trace from the task two trials ago (at n–2).

The retrieval probability—*p*(retrieval)—influences the speed of responding. If retrieval succeeds, the response is facilitated due to retrieval of elements of the previous episodic trace that prime performance on the current trial; if retrieval fails, the response is not facilitated, and thus responding is slower as it has to be performed via a slower, algorithmic, route (see Grange and Cross, 2015, for discussion).

#### RT distributions

To predict response time distributions, the mathematics of the Linear Ballistic Accumulator (Brown and Heathcote, 2008) was used. The LBA is a successful model of choice response time, allowing the modeling of correct and error RT distributions. The model assumes that, when presented with a task stimulus, evidence accumulates toward a retrieval threshold. Evidence for each response—typically simplified to the “correct” and “error” response—accumulates in a linear fashion until one of the accumulators reaches the response threshold. At this point, it is assumed that this response has been selected by the model.

The LBA model has several parameters. The mean rate of evidence accumulation is referred to as the *drift rate*, *v*; it is higher for the correct response than for the error response (which is typically set to 1–*v*). The drift rate for each accumulator can vary across trials, but is assumed to have a fixed mean rate. The drift rate on each trial is a random draw from a normal distribution with mean *v* and standard deviation *s*. The starting point of the accumulation process can vary uniformly between 0 and *A*. The height of the response threshold is governed by the parameter *b*. The time taken to perceptually encode stimuli and make a manual response is captured by a single non-decision time parameter *ter*.

The Upper portion of Figure 5 shows how the LBA is applied in the current context. (Note that only the correct accumulators are shown to avoid clutter.) The model only ever uses one of two accumulation processes: If retrieval is successful (with probability *p*[retrieval]), the model samples the current RT from a “successful,” “fast” accumulation process with a higher drift rate, *v*_*Fast*_; if retrieval fails (with probability 1 – *p*[retrieval]), the current RT is sampled from an “unsuccessful,” “slow” accumulation process with a lower drift rate, *v*_*Slow*_.

Thus, in this model, when TD is high, *p*(retrieval) is high, and as such more trials will be sampled from the fast accumulation process. When TD is low, so too will *p*(retrieval), and as such more trials will be sampled from the slow accumulation process. Intermediate TDs will have an intermediate *p*(retrieval), and as such their RT distributions will be a weighed mixture of the two base distributions (fast and slow), with the exact proportion governed by *p*(retrieval).

### Fitting the model

All modeling was performed on group-averaged data. Grange and Cross (2015) used the full mathematics of SIMPLE to obtain *p*(retrieved). To reduce the number of free parameters, in the current model *p*(retrieved) was treated as a “semi-fixed” parameter. To achieve this, the RCI-ratio was calculated for each RCI-sequence condition in Figure 2. Specifically, the RCI-ratio was 20 for n–2 to n–1 RCIs of 1000–50 ms; the ratio was 1 for RCIs of 1000–1000 ms and 50–50 ms; and the ratio was 0.05 for RCIs of 50–1000 ms. *p*(retrieved) was “semi-fixed” in the sense that it was fixed at *p*(retrieved) = 1 for ratios of 20 (i.e., perfect retrieval) and fixed at *p*(retrieved) = 0 for ratios of 0.05 (i.e., failed retrieval); *p*(retrieved) was free to vary for ratios of 1. Thus, *p*(retrieved) controls the relative contribution of the two RT distributions for intermediate RCI ratios. It is of course possible that even in the RCI-ratio of 20 some retrieval failures occur (and vice-versa for RCI-ratios of 0.05). However, for the purposes of this modeling work, I use the simplifying assumption described.

Separate drift rates were estimated for the “fast” and “slow” distributions—*v*_*Fast*_ and *v*_*Slow*_, respectively. (Note that drift rates for error response evidence accumulation is given as 1–*v*.) Also, each distribution had its own response threshold—*b*_*Fast*_ and *b*_*Slow*_^1^. All other parameters (i.e., *A*, *s*, and *ter*) took on identical values for all three ratio conditions.

Note therefore that this fit is rather ambitious. First, the model has to explain whole RT distributions for correct and error responses across all three conditions of RCI ratio. Also, the data for RCI-ratio = 1 is never explicitly modeled; rather, the model is fit to the RCI-ratio = 20 with a fast distribution and to RCI-ratio = 0.05 data with a slow distribution; the data for RCI-ratio = 1 is then estimated by a weighted contribution of the two distributions controlled only by *p*(retrieved).

**Figure 6:**
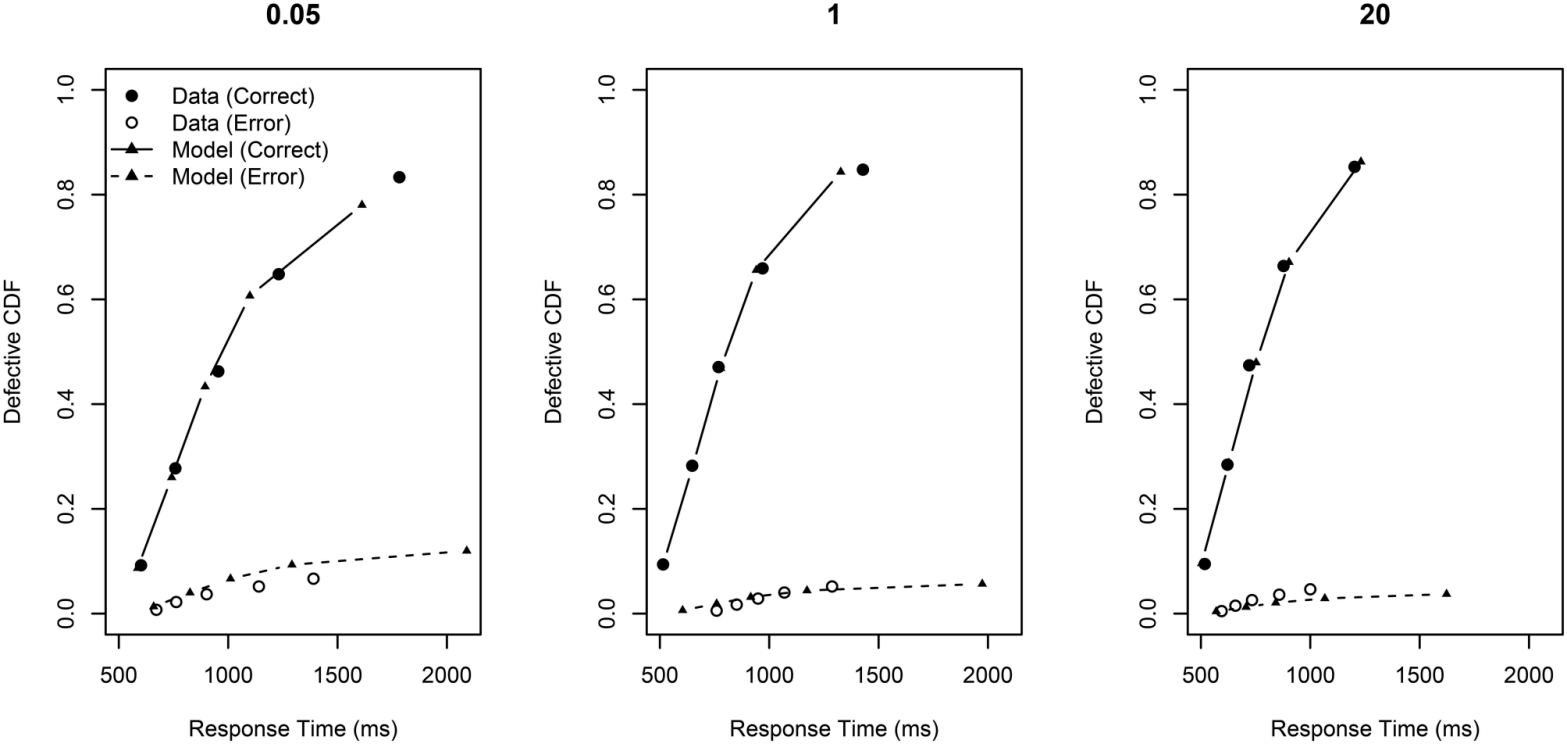
**Defective cumulative distribution functions for the behavioral data and the model fit for all RCI-ratios (0.05, 1, and 20) for correct and error RT distributions**.

The model was fitted to the data via a version of Quantile Maximum Probability Estimation (QMPE Heathcote et al., 2002), where the model is fit to observed frequencies between RT quantiles rather than individual RTs as per maximum likelihood (see Appendix C for details). This method was used due to the relatively low number of trials per subject, and the rather low error rate. The fits of the model to the behavioral data are shown in Figure 6. The best-fitting parameters are shown in Table 1.

**Table 1:** **Best fitting model parameters from the fit routine. Note that *p*(retrieved) refers to the probability of sampling from the fast distribution only for ratio = 1 data points**.

**Model parameter**	**Value**
*p*(*retrieved*)	0.759
*A*	718.14
*b*_*Fast*_	114.58
*b*_*Slow*_	179.96
*v*_*Fast*_	1.132
*v*_*Slow*_	0.893
*s*	0.44
*ter*	346.42

*See text for details*.

This Figure presents so-called “defective” cumulative distribution functions (CDFs). The data are sorted from fastest to slowest, and then quantile cut-off points are calculated for each subject according to a pre-specified set of quantiles $\vec{Q}$ (here $\vec{Q}=$ 0.1, 0.3, 0.5, 0.7, 0.9). The Figure plots the average RT for each quantile across subjects against the quantile value. The plot is a defective CDF in the sense that it also displays information about errors. Specifically, RT is plotted against $\vec{Q}$ × mean accuracy rather than just $\vec{Q}$. Perfect accuracy would thus be plotting RT against $\vec{Q}$. Error RT—shown in the Figure as open circles)—is error RT plotted against $\vec{Q}$ × (1 - mean accuracy).

The overall fit statistic of the model was QMPE = 330.12. The model fit to correct RT distributions is rather good; the model's prediction for mean RT gave *R*^2^ = 0.986. The model captures all of the main trends in the data: RT becomes facilitated as *p*(retrieved) increases (from left-to-right *p*(retrieved) = 0, *p*(retrieved) = 0.759, and *p*(retrieved) = 1). This improvement in RT is largely at the slower end of the RT distribution. Also, the height of the defective CDFs increases as *p*(retrieved) is increased, reflecting generally better accuracy. Importantly, the central plot shows a good fit of the model to the data, providing support that intermediate TDs are a mixture of two base distributions.

The fit to the error RT distributions is not as good as for the correct RT distributions; the model's prediction for mean RT gave *R*^2^ = 0.864. However, accuracy was generally very high in this experiment (indeed, participants were excluded for not achieving greater than 80% accuracy, and average accuracy was much higher). So, there were often few error RTs per quantile bin during the model fit, making accurate estimation difficult. However, despite the poor quantitative fit, the qualitative pattern seen in the behavioral data is generally reproduced: error RT becomes generally faster and less variable as ratio increases.

In sum, this mixture-model provides a good account of the behavioral data.

## General discussion

In this paper, I was interested in assessing a key prediction from the temporal distinctiveness account of RCI effects in task switching: namely, that all responses are governed by one of only two processes: a “fast” process when an episodic trace is successfully retrieved, and a “slow” process when episodic retrieval fails. This account makes a specific prediction that intermediate RCI-ratios should be a mixture-distribution of a fast RT distribution and a slow RT distribution.

This prediction was largely supported. In the first section, I provide evidence that the data support the fixed-point property of mixture distributions. In the second section I developed a mixture-distribution model that extends the model of Grange and Cross (2015) to explain whole RT-distributions. As the model is forced to only sample from one of two processes, finding agreement between behavioral data and model predictions provides support for the mixture-distribution assumption. Generally, this condition was met, with overall good fit of the model to correct RT distributions; the fit to the error distributions was not so clear, although the qualitative pattern was reproduced. The modeling in this paper presents a “proof of concept” of the mixture-distribution assumption rather than being designed to be a complete explanation of temporal distinctiveness effects in task switching, so the fit can be considered successful in this regard.

### Model parameters

The fit of the model to the data allowed estimation of a number of important parameters, which might shed some light on explaining performance in RCI-ratio experiments. Importantly, the drift rate was estimated to be higher in the RCI-ratio 20 condition (i.e., *v*_*Fast*_) than in the RCI-ratio 0.05 condition (*v*_*Slow*_). This suggests that successful retrieval of the target episodic trace leads to faster RTs due to a speed-up in evidence accumulation for a response. In addition, the response boundary parameter *b* was reduced when retrieval failed (i.e., *v*_*Slow*_). This raises the interesting possibility that when the cognitive system fails to retrieve the targeted episodic trace, it enters a more cautious mode of responding by raising response threshold *b*; this might be in some way to compensate for the reduced efficiency of evidence accumulation when retrieval fails (*v*_*Slow*_).

### Switch trials

Here it is important to note that a complete model of TD effects in task switching will need to account for performance on switch trials. Typically, RCI-ratio does not appear to affect switch RT (Horoufchin et al., 2011a,b) which is why the present focus has been on repetition RTs only. The model presented here can easily be extended to account for switch RT performance by adding new LBA parameters for switch trials (e.g., *v*_*Switch*_, *A*_*Switch*_, *b*_*Switch*_, and possibly *s*_*Switch*_ and *ter*_*Switch*_). As RCI-ratio does not typically affect switch trials, it can be assumed that retrieval always fails on switch trials, so *p*(*retrieved*) can be set to zero for all RCI-ratio conditions.

## Conclusion

The present paper sought to assess the evidence in favor of a key prediction of the TD hypothesis of RCI effects on task repetition trials in task switching, namely that repetition RTs are a mixture of only one of two processes. In sum, this paper has provided good support for the TD prediction, which provides more support for these theoretical accounts (Horoufchin et al., 2011a,b; Grange and Cross, 2015). This presents an important challenge to models of cognitive control during task switching which assume control systems work to prevent relevant task-sets decaying. Instead, loss of retrieval of task-sets appears to be well-explained by interference effects rather than time-based decay.

## Author contributions

The author confirms being the sole contributor of this work and approved it for publication.

## Author note

I am grateful to Chris Donkin for discussion regarding the Linear Ballistic Accumulator model. All raw data, analysis code, and model code are available to download at http://bit.ly/1TiPnEu.

### Conflict of interest statement

The author declares that the research was conducted in the absence of any commercial or financial relationships that could be construed as a potential conflict of interest.

## Footnotes

^1^Initial fits with just *v* varying between the fast and slow distributions did not fit the data as well as when *b* was also allowed to vary between conditions.

## Appendix A

### Method and results (grange, in revision)

The data presented in Figure 2 presents a portion of the overall data presented by Grange (in revision). Speficially, this data comes from the “categorization” condition. In this condition, 25 participants were presented with a 2 × 2 square grid. Two arrow cues either pointed up and down (presented above and below the grid, respectively) or left and right (presented to the left and to the right of the grid, respectively). Hundred milliseconds later, a stimulus appeared in one of the grid's cells. The stimulus could be an A or a 4, presented in either red or blue font. If the arrows pointed up and down, the participants had to judge whether the stimulus was an A or a 4; if the arrows pointed left and right, the participants had to judge whether the stimulus was red or blue. Participants responded using the “1” and the “9” keys on the numerical part of the keyboard; 1 corresponded to “letter” and “red” responses, and 9 corresponded to “number” and “blue” responses. Once a response was registered, the grid was cleared for an RCI of either 50 or 1000 ms (chosen randomly). The next task was chosen randomly. In total, participants were presented with 6 blocks of 64 trials, preceded by 16 practice trials.

The ANOVA summary table for this data are shown in Table A1.

**Table A1:** **ANOVA summary table for response time data from Grange (in revision)**.

**Source**	**df**	***F***	***p***	**$\eta_{g}^{2}$**
Sequence (S)	1, 24	118.94	<0.001	0.469
RCI (R)	1, 24	12.61	0.002	0.022
RCI-Change (RC)	1, 24	15.68	<0.001	0.015
S ^*^ R	1, 24	32.03	<0.001	0.024
S ^*^ RC	1, 24	4.95	0.036	0.002
R ^*^ RC	1, 24	12.26	0.002	0.021
S ^*^ R ^*^ RC	1, 24	18.3	<0.001	0.008

## Appendix B

### Overview of Grange and Cross (2015) model

The Grange and Cross (2015) model used the full mathematics of the SIMPLE (Brown et al., 2007) model to account for TD effects on task repetition trials. Distinctiveness is proportional to the *similarity* between a target trace and its neighbors in episodic memory. In this model, similarity is only influenced by the temporal domain. Similarity of episodic instances at n–1 (item *i*) and n–2 (item *j*) is given by

(A1) $$\begin{array}{c} \eta_{ij}={\left(\frac{T_{i}}{T_{j}}\right)}^{c}, \end{array}$$

where *T*_*x*_ refers to the temporal age of an item and *c* scales the similarity. Temporal age is calculated from the current time. So, with RCIs of 50–1000 ms, *T*_*i*_ would equal 1000 ms, and *T*_*j*_ would equal 1050 ms. Based on the target trace *i*'s similarity to its neighbors, its discriminability *D*_*i*_ can be determined. This refers to how isolated the target trace *i* is in episodic memory, and is given by

(A2) $$\begin{array}{c} D_{i}=\frac{1}{\sum_{j=1}^{n}\eta_{ij}}. \end{array}$$

As we are only considering the similarity between two items (*i* and *j*), and as an item's similarity to itself is 1, Equation A2 becomes

(A3) $$\begin{array}{c} D_{i}=\frac{1}{\eta_{ij}+1}. \end{array}$$

Given an item's discriminability, we can calculate its retrieval probability *p*(*R*_*i*_) at time of retrieval *t* by

(A4) $$\begin{array}{c} p(R_{i}|D_{i})=\frac{1}{1+e^{-s(D_{i}-thresh)}}, \end{array}$$

where *s* and *thresh* are free parameters which describe the slope of the transforming function and the threshold of retrieval, respectively. Given these equations, we can calculate the retrieval probability for each level of temporal distinctiveness in the behavioral experiment. It is assumed that if the trace is retrieved (with probability *p*[retrieved]) then response time will be a random draw from a fast RT distribution with mean μ_*fast*_; if retrieval fails (with probability 1−*p*[retrieved]), the response time will be a random draw from a slow RT distribution with mean μ_*slow*_. Thus, mean RT for each level of TD is given by the RT-mixture equation

(A5) $$\begin{array}{c} RT=p(retrieved)\times \mu_{Fast}+[1-p(retrieved)]\times \mu_{slow}. \end{array}$$

## Appendix C

### Mixture-distribution model details

This section describes the details of the mixture-distribution model. The model extends the mathematics of the linear ballistic accumulator (Brown and Heathcote, 2008) for a unitary process (i.e., not a mixture model), which allows calculation of the defective probability density function of one response accumulator reaching retrieval threshold before the accumulator for the second response does. I first describe the mathematics of the LBA for a unitary process before describing how this can be extended to a mixture model.

#### LBA for unitary process

For a unitary process, to calculate the defective PDF of response option *i* reaching threshold first (before *j*), one first needs the cumulative distribution function (CDF) for *i* at time *t*, *F*_*i*_(*t*), given the model parameters *v*, *A*, *s*, *b*, and *t* = *t* − *ter*), is given by

(B1) $$\begin{array}{c} \begin{array}{l} F_{i}(t)=1+\frac{b-A-tv_{i}}{A}\Phi \left(\frac{b-A-tv_{i}}{ts}\right)-\frac{b-tv_{i}}{A}\Phi \left(\frac{b-tv_{i}}{ts}\right)\\ \;+\frac{ts}{A}\phi \left(\frac{b-A-tv_{i}}{ts}\right)-\frac{ts}{A}\phi \left(\frac{b-tv_{i}}{ts}\right), \end{array} \end{array}$$

(where Φ refers to the normal distribution's CDF). Then one needs the probability density function (PDF) of *i* at time *t*, *f*_*i*_(*t*) given by

(B2) $$\begin{array}{c} \begin{array}{l} f_{i}(t)=\frac{1}{A}[-v_{i}\Phi (\frac{b-A-tv_{i}}{ts})+s\phi (\frac{b-A-tv_{i}}{ts})\\ \;+v_{i}\Phi (\frac{b-tv_{i}}{ts})-s\phi (\frac{b-tv_{i}}{ts})] \end{array} \end{array}$$

where ϕ refers to the normal distribution's density. Considering two accumulators *i* and *j* (e.g., correct and error response, respectively), the defective PDF for *i* at time *t* is given by

(B3) $$\begin{array}{c} {\text{PDF}}_{i}(t)=f_{i}(t)\prod_{j\neq i}\left(1-F_{j}(t)\right). \end{array}$$

#### Modeling mixture-distributions

Extending this to a mixture-distribution model is as follows. If we denote the fast accumulation process as *Fast* and the slow accumulation process as *Slow*, recall that mean RT can be predicted by

(B4) $$\begin{array}{c} RT=p\mu_{Fast}+(1-p)\mu_{Slow}. \end{array}$$

Extending this mixture-distribution model to a likelihood function from the LBA, we can now obtain the PDF for response *i* (rather than response *j*) at time *t* by

(B5) $$\begin{array}{c} \begin{array}{l} {\text{PDF}}_{i}=\left[p\cdot f_{Fast[i]}(1-F_{Fast[j]})\right]+\\ \;\left[(1-p)\cdot f_{Slow[i]}\right](1-F_{Slow[j]}) \end{array} \end{array}$$

#### Model fit details

The model was fit to the data via a variant of the QMPE method (Heathcote et al., 2002). Specifically, the average quantile cut-off values were found for correct and error responses for each condition and accuracy response. The quantiles used were 0.1, 0.3, 0.5, 0.7, and 0.9. So, RTs for each each quantile value were stored in the vector $\vec{Q}$ with length *q* such that $\vec{Q}=$ *RT*_0.1_ …*RT*_0.9_. The number of RTs in each bin (i.e., in the range 0–$\vec{Q}$–1) were stored in the vector $\vec{N}$ with length *n*. Then, the defective cumulative distribution function *F* at time *t* = *t*−*ter*, *F*(*t*)^2^, given model parameters $\vec{\theta }$ was calculated, and multiplied by the number of trials within that bin to obtain the QMPE estimate

(B6) $$\begin{array}{c} \begin{array}{l} QMPE\left(\vec{\theta }|\vec{Q},\vec{N}\right)\propto ln\left[F\left(Q_{1}:\vec{\theta }\right)-F\left(0:\vec{\theta }\right)\right]N_{1}\\ \;\times ln\left[F\left(Q_{i}:\vec{\theta }\right)-F\left(Q_{i-1}:\vec{\theta }\right)\right]N_{i}\\ \;\times \ldots \\ \;\times ln\left[F\left(\infty :\vec{\theta }\right)-F\left(Q_{q}:\vec{\theta }\right)\right]N_{n}, \end{array} \end{array}$$

where *ln* is the natural logarithm.

This was performed for each RCI-ratio condition for correct and error responses, and the likelihoods for each were summed. The fit routine aimed to find the best-fitting parameters that minimized the negative summed likelihood; the Nelder-Mead method using R's optim function was used to fit the model. Starting values for the parameter search utilized the starting heuristics recommended by Donkin et al. (2011).
